# Characteristics of reproductive tract infections caused by common pathogens among the outpatients of reproductive medicine center in Putian: retrospective study

**DOI:** 10.1186/s12879-024-09180-9

**Published:** 2024-03-14

**Authors:** Jiancheng Zeng, Tingli Wu, Laiping Wang, Liumin Yu, Hua Lin, Zhanfei Chen

**Affiliations:** 1 The Affiliated Hospital of Putian University, Putian University, No.999 Dongzhen East Road, Licheng District, Putian, Fujian China; 2 Comprehensive Technology Service Center of Quanzhou Customs, Inspection and Quarantine Bureau Building, South Section of Citong East Road, Quanzhou, Fujian China; 3 Key Laboratory of Medical Microecology (Putian University), Fujian Province University, No.1133 Xueyuan Middle Street, Chengxiang District, Putian, Fujian China

**Keywords:** Putian city, Fujian province, Reproductive medicine centre, STD pathogens, Infertility, Age

## Abstract

**Background:**

This study aims to explore the infection and age distribution of *Ureaplasma urealyticum* (UU), *Chlamydia trachomatis* (CT), *Neisseria gonorrhoeae* (NG) and *Herpes simplex virus type II* (HSV II) among the outpatients of Reproductive Medicine Center in Putian, Fujian Province to provide a clinical basis for the early diagnosis and treatment of various reproductive tract diseases and infertility in this region.

**Methods:**

A total of 1736 samples of secretions and exfoliated cervical cells were collected from the outpatients of the Reproductive Medicine Center of the Affiliated Hospital of Putian University from December 2021 to April 2023. The infections of UU, CT, NG and HSVII were detected by real-time fluorescence polymerase chain reaction (PCR), and the infection statuses of the patients with different genders, ages and diagnoses were analysed.

**Results:**

Among the 1736 patients, 611 were male and 1125 were female. The male patients had higher UU infection rate but lower HSV II infection rate than the female patients. No significant difference in CT and NG infection rates was observed between the genders. The CT infection rate gradually decreased with the increase in the age. The difference in UU, NG and HSV II infection rates among the different age groups was not statistically significant. For UU infection, the male infertile patients had the highest rate of 37.72% (172/456). Meanwhile, the differences in CT, NG and HSV II infection rates among the different diagnosis groups were not statistically significant. Among the male and female infertile patients, the CT infection rate was the highest in the 21–25 years of age group at 11.11% (2/18) and 9.47% (9/95), respectively. No statistically significant difference in UU, CT, NG and HSV II infection rates was observed among the different age groups of patients diagnosed in relation to the family planning guidance and between the male and female patients with other diagnoses results.

**Conclusions:**

This study showed that UU was the most frequently identified pathogen in infertile men in Putian, Fujian Province. The CT infection rate was the highest in people under 20 years old, and the infection showed a tendency toward young individuals. Therefore, the publicity of sexual health knowledge must be strengthened, and the prevention and treatment of venereal diseases among young and middle-aged people must be improved. Moreover, the pathogen infection is related to infertility to a certain extent, which is conducive to clinical diagnosis and treatment.

## Introduction

Sexually transmitted diseases (STDs) refer to a group of infectious diseases that are transmitted through sexual contact, cause urogenital lesions and spread through the blood to invade all tissues and organs of the body, even leading to infertility in serious cases [1, 2]. In the recent decade, the STD spectrum has widened, and more than 20 diseases have been included. For example, *Chlamydia trachomatis* (CT) can cause urethritis, neonatal conjunctivitis, cervicitis and infertility; *Ureaplasma urealyticum* (UU) is the main cause of nongonococcal urethritis; a variety of bacteria, such as *Gonococcus*, *Enterobacter* and *Brucella*, can be transmitted through sexual behaviour; *Herpes simplex virus* (HSV) is a high risk factor of genital herpes; and *Spirochaeta pallidum* is the pathogen of syphilis [3, 4].

The global incidence of sexually transmitted infections (STIs) remains high, with more than 1 million STIs acquired every day [5]. In developed countries, such as the United States, the rates of *Gonorrhoea*, *Chlamydia* and *Syphilis* have increased, and those of HSV I and HSV II have decreased [6]. In China, the infection situation of STD pathogens is also not optimistic, and *Ureaplasma*, *Neisseria gonorrhoeae* (NG), *Chlamydia* and other pathogens have reached a certain scale of infection [7–9]. NG, CT, UU and HSV II cause STDs and are closely related to infertility [5]. The STIs caused by NG and CT are the common causes of vaginitis that can lead to tubal factor infertility, one of the most common causes of infertility that accounts for 30% of female fertility problems [9–11]. UU infection can affect the morphology and quality of male sperms and therefor is an important cause for male infertility [12, 13]. UU and CT are important pathogens of nongonococcal urethritis. The sexual behaviour may prompt genital tract inflammation in male and female, which is an important factor of infertility [13, 14]. Genital herpes caused by HSV II easily recurs and can also infect newborns through placenta and birth canal, resulting in congenital infection in newborns.

Some patients infected with UU, CT, NG or HSV II may not show evident clinical symptoms [6], leading to increased difficulty for the early diagnosis, treatment and control of disease transmission. This study analysed 1736 outpatients in the Reproductive Medical Center of the Affiliated Hospital of Putian University. Real-time fluorescent polymerase chain reaction (PCR) was used to detect UU, CT, NG and HSV II pathogens and analyse the infections of the patients of different genders, ages and diagnoses. A laboratory basis for clinical diagnosis and treatment was provided.

## Materials and methods

### Study population

After the analysis of the sample information of the patients who visited the outpatient clinic of Reproductive Medicine Center of the Affiliated Hospital of Putian University from December 2021 to April 2023 for a variety of disease causes, a total of 1736 cases were included. Statistical analysis was performed on the nucleic acid detection results of UU, CT, NG and HSV II infection in the samples from 611 male patients and 1125 female patients within an age range of 16–61 (31.36 ± 4.93) years. A total of 1127 secretion samples and 609 exfoliated cervical cell samples, 456 cases of clinically diagnosed male infertility and 909 cases of clinically diagnosed female infertility, and 221 cases related to family planning guidance and 150 cases of other diagnoses were included in this study. The complicated factor of fallopian tubes and uterus were grouped into “Other diagnosis”. The inclusion criteria were as follows: (1) women who did not receive vaginal medication or take anti-inflammatory drugs within two weeks and (2) women who tested positive for UU, CT, NG and HSV II pathogens for the first time. The exclusion criteria were as follows: (1) women with incomplete information and secondary examination and (2) women who had chronic diseases and needed long-term medication. The Ethics Committee of the Affiliated Hospital of Putian University approved this study (No. 2023042), and all procedures were carried out in strict accordance with the *Helsinki Declaration*.

### Sample collection and analysis

For genomic DNA extraction, the samples of secretion or exfoliated cervical cells of the patients were collected in accordance with the instructions. The samples were then thoroughly oscillated and mixed. Volume 320 µL of liquid was absorbed for nucleic acid extraction. Afterward, the DNA (2 µL) was mixed with an amplification reagent. The amplification tube was covered tightly and centrifuged instantaneously. Real-time fluorescent quantitative PCR was used for amplification reaction, and all procedures were carried out in strict accordance with the instructions [15]. Each amplification required negative and positive references for quality control.

### Main instruments and test reagents

The main instruments used in this study included a sample oscillator (Jiangsu Xinkang Medical Instrument, XK80-A), a high-speed freezing centrifuge (Anhui USTC Zonkia Scientific Instrument, HC-3018R), an automatic nucleic acid extractor (Guangzhou Daan Gene, Smart 32) and a fluorescent quantitative PCR (Applied Biosystems, QuantStudio 5).

A nucleic acid extraction reagent (Guangzhou Daan Gene, DA0623) was used for nucleic acid extraction. Nucleic acid test kits for UU (Guangzhou Daan Gene, DA0080-DA0083), CT (Guangzhou Daan Gene, DA0070-DA0073), NG (Guangzhou Daan Gene, DA0060-DA0063) and HSV II (Guangzhou Daan Gene, DA1510) were used to detect the four pathogens, and the operation and result interpretation were carried out in strict accordance with the kit instructions.

### Statistical analysis

SPSS 26.0 software and GraphPad Prism 7.0 software were used for data analysis. The counting data were expressed as case number or composition ratio. The χ^2^ test was used to compare the infection rates among different groups. *P* < 0.05 was considered to be statistically significant.

## Results

### Analysis of UU, CT, NG and HSV II infections

**Fig. 1:**
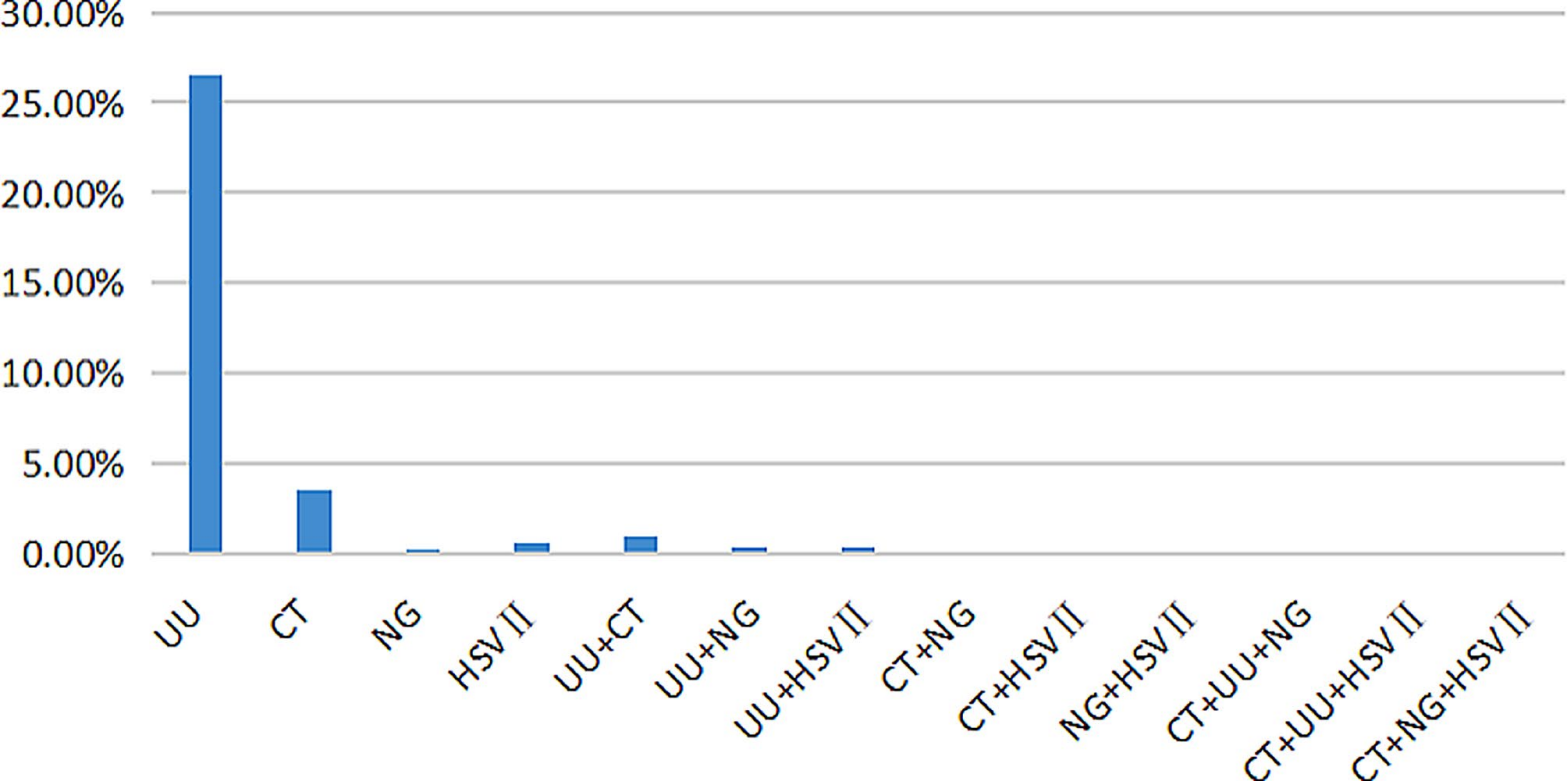
Analysis of UU, CT, NG and HSV II infections

Among the 1736 patients, 611 were male and 1125 were female. The samples of all the male patients were secretion samples, and those of the female patients included 516 samples of secretions and 609 exfoliated cervical cell samples. Laboratory examination showed that the infection rate was 26.50% (460/1736) for UU, 3.57% (62/1736) for CT, 0.23% (4/1736) for NG and 0.58% (10/1736) for HSV II. In particular, 17 patients (0.98%) had CT + UU mixed infection, 2 (0.12%) had UU + NG mixed infection and 2 (0.12%) had UU + HSV II mixed infection. No multiple infections such as CT + NG, CT + HSV II, NG + HSV II and CT + UU + NG were detected (Fig. 1).

### Comparison of the infection rates of various pathogens between male and female patients

The UU, CT, NG and HSV II infection rates in male patients were 35.68% (218/611), 3.11% (19/611), 0.33% (2/611) and 0% (0/611), respectively, and those in female patients were 21.51% (242/1125), 3.28% (43/1125), 0.18% (2/1125) and 0.89% (10/1125), respectively. The UU infection rate in men was higher than that in women, and the HSV II infection rate in women was higher than that in men; the difference was statistically significant (*P* < 0.05). However, the difference in the CT and NG infection rates among the patients of different genders was not statistically significant (*P* > 0. 05) (Table 1).

**Table 1:** Male and female infection rates for various pathogens

Gender	No.	UU (%)	CT (%)	NG (%)	HSVII (%)
Male	611	218 (35.68)	19 (3.11)	2 (0.33)	0
Female	1125	242 (21.51)	43 (3.28)	2 (0.18)	10 (0.89)
Total no.	1736	460 (26.50)	62 (3.57)	4 (0.23)	10 (0.58)
χ^2^		40.890	0.584	0.385	5.463
**P**		**<0.05**	**>0.05**	**>0.05**	**<0.05**

### Comparison of the infection rates of various pathogens among patients of different age groups

The CT infection rates differed statistically significantly among the age groups (*P* < 0.05). The number of patients positive for CT was the highest among those under the age of 20 years (11.11%), followed by those between the ages of 21–25 years (8.00%), and was the smallest among those aged above 40 years (2.02%) (*P* < 0.05). UU infections were highly positive, but the difference had no statistical significance (*P* > 0. 05) probably because UU is a normal flora in the female reproductive tract and has the characteristics of universal infectivity [16]. The difference in the NG and HSV II infection rates among the patients of different age groups was not statistically significant (*P* > 0. 05) (Table 2).

**Table 2:** Comparison of the infection rates of various pathogens among patients of different age groups

Age group (y)	No.	UU (%)	CT (%)	NG (%)	HSVII (%)
≤ 20	9	2 (22.22)	1 (11.11)	0	0
21–25	157	45 (28.66)	14 (8.92)	1 (0.64)	2 (1.27)
26–30	577	134 (23.22)	21 (3.64)	2 (0.35)	4 (0.69)
31–35	691	191 (27.64)	17 (2.46)	1 (0.14)	2 (0.29)
36–40	237	69 (29.11)	7 (2.95)	0	2 (0.84)
>40	65	19 (29.23)	2 (3.08)	0	0
χ ^2^		5.184	17.305	2.407	3.190
P		>0.05	**<0.05**	>0.05	>0.05

### Analysis of CT, NG, UU and HSV II infections according to clinical diagnoses

**Fig. 2:**
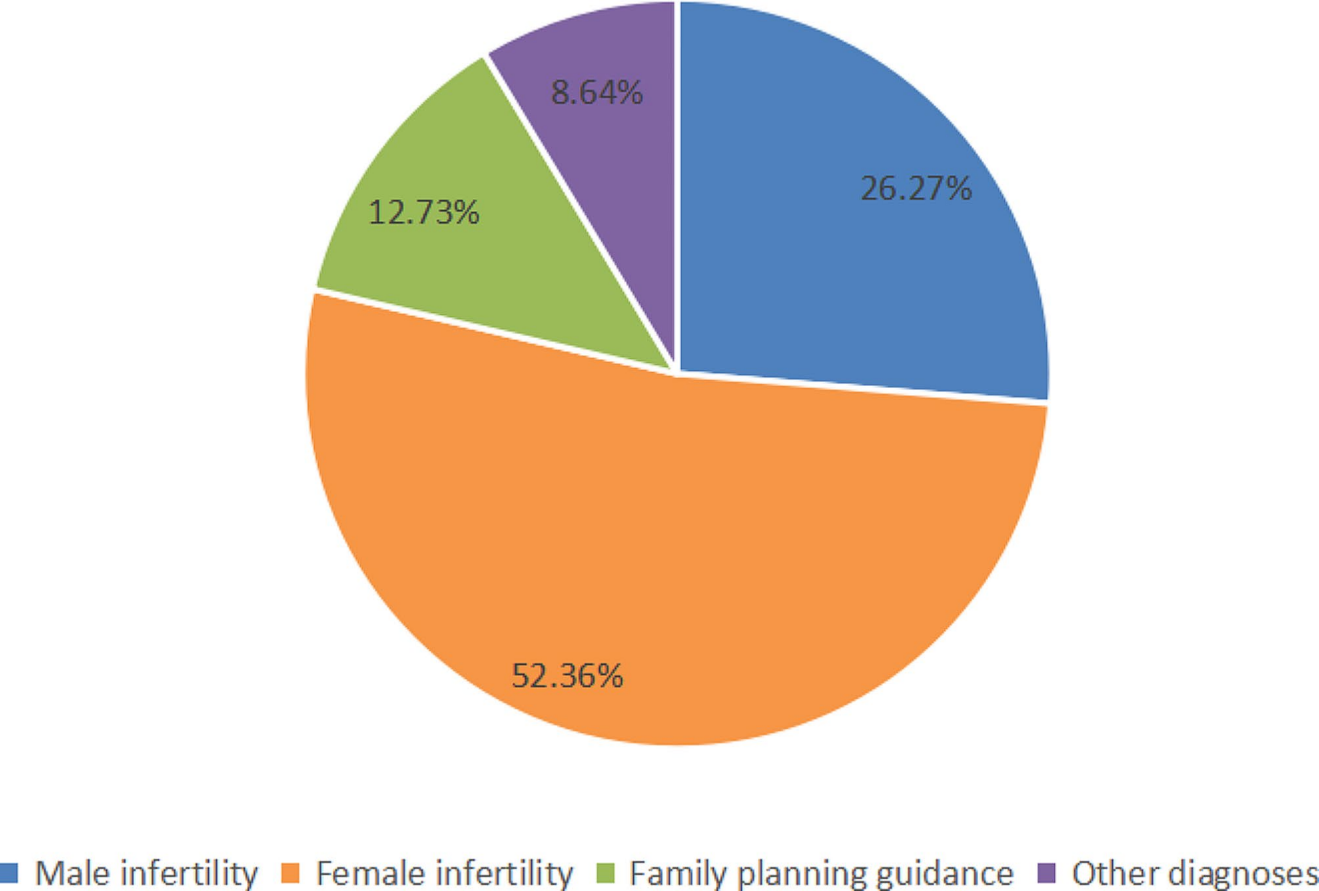
Proportion of different diagnosis groups

All 1736 cases were divided into the following four groups according to different clinical diagnoses: male infertility, female infertility, family planning guidance and other diagnoses (Fig. 2).

The difference in the UU infection rate among the different clinical diagnosis groups was statistically significant (*P* < 0.05). The highest UU infection rate occurred in the patients with male infertility (37.72%), followed by that in the patients related to family planning guidance (27.60%). The number of positive cases of CT, NG and HSV II was small among the different clinical diagnosis groups, and the infection rates did not significantly differ (*P* > 0.05) (Table 3).

**Table 3:** Comparison of the infection rates of pathogens among different clinical diagnosis groups

Clinical diagnoses	No.	UU (%)	CT (%)	NG (%)	HSVII (%)
Male infertility	456	172 (37.72)	10 (2.19)	1 (0.22)	0
Female infertility	909	199 (21.89)	34 (3.74)	2 (0.22)	8 (0.88)
Family planning guidance	221	61 (27.60)	11 (4.98)	0	1 (0.45)
Other diagnoses	150	28 (18.67)	7 (4.67)	1 (0.67)	1 (0.67)
χ ^2^		44.243	4.382	1.759	4.190
P		**<0.05**	>0.05	>0.05	>0.05

### Comparison of the infection rates of various pathogens in male infertile patients among different age groups

Among the 1736 patients, 456 (26.27%) were diagnosed with male infertility. Laboratory examination showed that the infection rate was 37.72% (172/456) for UU, 2.19% (10/456) for CT, 0.22% (1/456) for NG and 0% (0/456) for HSV II. The highest number of patients positive for UU infection was among those aged 31–35 years at 218, accounting for 47.81%. The CT infection rate gradually decreased in the patients aged above 21–25 years (*P* < 0.05). UU infections were highly positive, but the difference was not statistically significant (*P* > 0.05). The CT, NG and HSV II infection rates did not differ statistically significantly among the patients of different ages (Table 4).

**Table 4:** Comparison of the infection rates of various pathogens among the male infertility patients of different age groups

Age group (y)	No.	UU (%)	CT (%)	NG (%)	HSVII (%)
≤ 20	0	0	0	0	0
21–25	18	8 (44.44)	2 (11.11)	0	0
26–30	122	41 (33.61)	2 (1.64)	0	0
31–35	218	83 (38.07)	3 (1.38)	1 (0.46)	0
35–40	73	28 (38.36)	3 (4.11)	0	0
>40	25	12 (48.00)	0	0	0
Total no.	456	172 (37.72)	10 (2.19)	1 (0.22)	0
χ ^2^		2.374	11.507	1.094	-
P		>0.05	**<0.05**	>0.05	-

### Comparison of the infection rates of various pathogens among the female infertile patients of different age groups

Among the 1736 patients, 909 (52.36%) were diagnosed with female infertility and provided 532 exfoliated cervical cell samples (58.53%) and 377 samples of secretions (41.47%). Laboratory examination showed that the infection rate was 21.98% (199/909) for UU, 3.74% (34/909) for CT, 0.22% (2/909) for NG and 0.88% (8/909) for HSV II. The CT infection rate was the highest in the group of patients aged 21–25 years (9.47%) and showed a continuously decreasing trend with age (*P* < 0.05). The UU infection rate was high but had no statistical significance (*P* > 0.05) possibly because UU is a normal flora in the female reproductive tract and has universal infectivity [16], so the results have no statistical significance despite the high positive detection rate. No statistically significant difference in the NG and HSV II infection rates was observed among the different age groups (*P* > 0.05) (Table 5).

**Table 5:** Various pathogens infection rates in female infertility patients of different age groups

Age group (y)	No.	UU (%)	CT (%)	NG (%)	HSVII (%)
≤ 20	2	1 (50.00)	0	0	0
21–25	95	24 (25.26)	9 (9.47)	1 (1.05)	2 (2.11)
26–30	325	69 (21.23)	13 (4.00)	1 (0.31)	3 (0.92)
31–35	365	74 (20.27)	11 (3.01)	0	2 (0.55)
36–40	112	30 (26.79)	1 (0.89)	0	1 (0.89)
>40	10	1 (10.00)	0	0	0
Total no.	909	199 (21.98)	34 (3.74)	2 (0.22)	8 (0.88)
χ ^2^		4.593	12.282	4.192	2.210
P		>0.05	**<0.05**	>0.05	>0.05

### Comparison of the infection rates of various pathogens among the patients related to family planning guidance of different age groups

Among the 1736 patients, 221 (12.73%) were diagnosed to be related to family planning guidance, including 122 male patients (55.20%) with secretion samples and 99 female patients (44.80%) with 38 exfoliated cervical cell samples (38.38%) and 61 samples of secretions (61.62%). Laboratory examination showed that the infection rate was 27.60% (61/221) for UU, 4.98% (11/221) for CT, 0% (0/258) for NG and 0.45% (1/221) for HSV II. A number of positive UU cases were recorded, and the highest infection rate occurred in the people aged 31–35 years (36.92%); however, the difference was not statistically significant (*P* > 0.05). The number of positive CT, NG and HSV II cases among the different clinical diagnosis groups was extremely small, and the infection rates did not differ statistically significantly between the groups (*P* > 0.05) (Table 6).

**Table 6:** Comparison of the infection rates of various pathogens among patients related to family planning guidance of different age groups

Age group (y)	No.	UU (%)	CT (%)	NG (%)	HSVII (%)
≤ 20	2	0	0	0	0
21–25	34	10 (29.41)	2 (5.88)	0	0
26–30	80	18 (22.50)	5 (6.25)	0	1 (1.25)
31–35	65	24 (36.92)	1 (1.54)	0	0
36–40	29	7 (24.14)	3 (10.34)	0	0
>40	11	2 (18.18)	0	0	0
Total no.	221	61 (27.60)	11 (4.98)	0	1 (0.45)
χ ^2^		5.499	4.640	-	1.771
P		>0.05	>0.05	-	>0.05

### Comparison of the STD infection rates between the male and female patients with other diagnoses

Among the 1736 patients, 150 (8.64%) had other diagnoses, including 33 male patients (22.00%) with secretion samples and 117 female patients (78.00%) with 39 exfoliated cervical cell samples (33.33%) and 78 secretion samples (66.67%). Laboratory examination showed that the infection rate was 18.67% (28/150) for UU, 4.67% (7/150) for CT, 0.67% (1/150) for NG and 0.67% (1/150) for HSV II. A number of male and female patients were infected by UU, CT, NG or HSV II pathogens, and the highest infection rate of UU (19.66%) occurred in the female patients. However, the infection rates of the four pathogens did not differ statistically significantly among the groups (*P* > 0.05) (Table 7).

**Table 7:** Comparison of sexuality rate between male and female patients with other diagnoses

Gender	No.	UU (%)	CT (%)	NG (%)	HSVII (%)
Male	33	5 (15.15)	1 (3.03)	1 (3.03)	0
Female	117	23 (19.66)	6 (5.13)	0	1 (0.85)
Total no.	150	28 (18.67)	7 (4.67)	1 (0.67)	1 (0.67)
χ ^2^		0.344	0.255	3.567	0.284
P		>0.05	>0.05	>0.05	>0.05

## Discussion

This study examined 1736 samples from the outpatient clinic of the Reproductive Medicine Center of the Affiliated Hospital of Putian University from December 2021 to April 2023. The results showed that the infection rate was 26.50% for UU, 3.57% for CT, 0.23% for NG and 0.58% for HSV II. The infection was mainly caused by a single pathogen, and co-infection was rare. The overall infection rate of NG pathogens was relatively low, and there was no statistical significance. However, three pathogens of UU, CT and HSVII have attracted our attention.

Studies in different regions of China suggested that *Ureaplasma* has a high prevalence [7]. The UU infection rate (26.50%) was significantly higher than that of the other three pathogens, which was consistent with the situation in Southwest China [17], Haikou [18] and Taizhou [19]. However, there were differences in UU infection rates in different countries. An Italian observational multicenter study revealed that the UU infection rate was only 9% [20]. A meta-analysis in Iran showed that the infection rate was 17.53% [21]. The UU infection rates in Moscow (29.1%) [22] and northeastern Romania (28.46%) [23] were similar to the results of this study. UU has a high risk of infection for male patients, which is different from the reports in Anhui Province [24] and Beijing City [8]、Iran [21]. UU infection was found in the six age groups, >40 and 36–40 ranked in the top two, and the infection rate ≤ 20 was the lowest. The results of this study were consistent with the highest UU infection rate in the 21–50 age group in China [25]. The relationship between infection rate and age was slightly different in different provinces of China. For example, the age groups most affected in Anhui Province were 21–30 years old and 31–40 years old [24]. Compared to other countries, women among the ages of 30 and 35 were the most affected in northeast Romania, followed by those aged 25 to 30. Serbia, Türkiye and Italy have the highest infection rates among 16–39 years old. This difference was related to the geographical and cultural characteristics of the different populations studied [26–28].

Among the outpatients of the Reproductive Medical Center, 78.62% were diagnosed with male or female infertility. The UU infection rate of the patients of different diagnosis groups from high to low was 37.72% in the male infertility group, 27.60% in the family planning guidance related group, 21.89% in the female infertility group and 18.67% in the group of other diagnoses. The difference was statistically significant, indicating that UU infection was closely related to male infertility. The UU infection rate of infertile people in China was significantly higher than the global average [29]. UU infection was closely related to male infertility in China [30]. The results of this study were consistent with this view. UU can parasitize in the urogenital tract and enter the seminal tract, seminal vesicle and testis through sexual behaviour, causing inflammation and damaging sperm quality [31, 32].

*Chlamydia* is one of the most common STIs in the world. The WHO recorded 129 million new cases of CT infection by 2020 [33]. The overall CT infection rate in this study was 3.57%, slightly higher than the global infection rate (2.9%) [34]. In detail, the infection rate of CT in this area was lower than that of Americas (4.5%), but higher than that of Europe (2.7%), Africa (2.6%), Western Pacific (2.6%) and South-East Asia (0.8%) [34]. CT infection rates in Belgium (1.54%) [35], Sao Paulo, Brazil (2.2%) [36] and the United States (2.35%) [37] were also low, while those in Tanzania (12.9%) [38] were not optimistic.

The proportion of CT infection is particularly high in young people [39]. The results of this study also support this view. The risk of CT infection in people aged under 25 years is higher than that in people of other age groups, and its rate decreases with age. CT infection shows a trend among young people, which is similar to the situation in Haikou region [18]. Globally, the situation was very similar. CT infections in young people aged 15–24 accounted for 2/3 of the total number of new infections [40]. Similar results have been reported in Russia [41], Sao Paulo, Brazil [36] and Germany [42]. Based on the mode of transmission of CT pathogens, we need to pay more attention to the infection of sexually active women under the age of 25 years [40] and to strengthen the promotion of health science. Among the patients with male or female infertility, the highest CT infection rates occurred in people aged 21–25 years. The trend of CT infection in younger people is also an important challenge to reproductive health [43, 44].

HSV type II mainly causes genital ulcer disease. It can increase the risk of HIV infection in men and women and can be transmitted to the foetus during pregnancy which is very harmful to the health of newborns [45–47]. The high seroprevalence of HSV II was found in Zhejiang and Shanghai [48, 49]. However, HSV II has a low infection rate (0.58%) and is mainly seen in female Putian patients, similar to that reported in Shandong Province [50]. There were regional differences in HSV II infection rates around the world. Infection rates in the United States [51] and Europe [52] have declined recently. Most HSV II infections were in Africa [53, 54], where the public health burden was greater than in other places.

This study analysed the infection of STD pathogens among the outpatients in the Reproductive Medical Center to help understand the epidemiological trend of the whole region. However, some insufficiencies must be acknowledged. Firstly, the data were derived from the past 2 years, and long-term dynamic observations are lacking. Dynamic analysis for at least 5–10 consecutive years is important for understanding the regional epidemiology; this will be our future research direction. Secondly, the sample size is still limited, and the results may not accurately reflect the region’s overall situation. In our future research, we will continue to collect samples and even adopt multi-centre joint research. In this study, the patients who were tested for venereal pathogens in this centre were mainly concentrated in the reproductive medical centre. The fields of reproductive medicine, such as venereal pathogens and infertility fields, are closely related [2, 9], which is an innovative perspective for this study.

The number of people infected with STD is annually increasing, and the trend of infection leans toward younger people. Especially in cities with great economic development, young people are open with regard to sexual attitudes and behaviours, but their awareness of self-protection is still insufficient. Therefore, health departments and medical institutions should actively promote publicity and education on sexual health, advocate correct sexual behaviours and encourage regular physical examination among young groups and premarital examination of special people groups to prevent the spread of STDs from the source. These actions will be of great significance to the physical and mental health of young people groups, the growth rate of newborns in China and the prevention of population aging in China.

## Conclusions

This study systematically analysed the common pathogens of reproductive tract infection in Putian region and achieved some important results. The UU infection rate in men is higher than that in women. The CT infection rate gradually decreases with age and shows a trend toward younger people. In addition, UU infection is an important factor for male infertility. The infection rates of the other two pathogens are relatively low, but they still have an influence on patients and require attention. Reproductive health is very important globally, and science popularization should be strengthened, especially for young people.

## Data Availability

The datasets used or analysed during the current study are available from the corresponding author on reasonable request.

## Abbreviations

STD: Sexually transmitted diseases
NG: *Neisseria gonorrhoeae*
CT: *Chlamydia trachomatis*
UU: *Ureaplasma urealyticum*
HSV II: *Herpes simplex virus type II*
